# Status of nutritional literacy in adolescents in the semi-rural area in Turkey and related factors

**DOI:** 10.1017/S1368980021002366

**Published:** 2021-08

**Authors:** Çağla Ayer, Ahmet Ergin

**Affiliations:** 1 İzmir Kâtip Çelebi University, Faculty of Health Sciences, Department of Nutrition and Dietetics, 35620Izmir, Turkey; 2 Pamukkale University, Faculty of Medicine, Department of Public Health and Department of Pediatrics, Department of Social Pediatrics, Denizli, Turkey

**Keywords:** Nutrition, Nutrition literacy, Adolescent nutrition, Adolescent nutrition literacy, Nutrition and dietetics, Nutrition habits

## Abstract

**Objective:**

To determine the status of nutritional literacy and its affecting factors among the adolescents who are in the 9th grade in Çivril, in Denizli province, in Turkey.

**Design:**

This is a cross-sectional study that determines the sociodemographic characteristics, nutritional habits, nutritional behaviour, nutritional literacy status of adolescents and affecting factors.

**Setting:**

Denizli, Turkey.

**Participants:**

We included 523 adolescents in ninth grade in this study.

**Results:**

Half (49·7 %) of the participants were female; 47 %, in normal BMI; and 68·1 %, non-smokers. The mean (sd) Adolescent Nutrition Literacy Scale score was 67·6 (sd 7·9). Nutrition literacy status was related to mothers’ education level (*P* 0·021); health perceptions (*P* 0·008); positive body perception (*P* 0·032); unhealthy food consumption status (*P* 0·017); information barriers (undecided about effort for information gathering (*P* 0·026), undecided about the difficulty of understanding information (*P* 0·042) and thinking it is difficult to understand (*P* 0·003)), trust in nutrition, diet information sources (nutrition and diet expert, dietitian trusting (according to others) (*P* 0·001), nutrition and diet expert, dietitian neutral to trust (compared with others) (*P* 0·011) and trust in textbooks (*P* 0·023)).

**Conclusions:**

The level of nutrition literacy status of participants was moderate. It is important to carry out interventions to increase the education level of women, positive body perceptions and general health perceptions of adolescents and to remove information barriers related to nutrition.

Adolescence offers an opportunity window to ensure a successful transition to adulthood. The nutritional status and eating behaviours acquired during this stage of life have important effects on the health and welfare of the adolescent as well as the intergenerational health outcomes^(1)^.

Health literacy (HL) ‘entails people’s knowledge, motivation and competences to access, understand, appraise and apply health information in order to make judgments and take decisions in everyday life concerning health care, disease prevention and health promotion to maintain or improve quality of life throughout the life course’ and has been a subject of growing interest in the last decades^(2)^. Optimising HL can help in improving health and well-being and reducing health inequities^(3)^. Food, nutrition and media are found under the umbrella of HL^(4)^. Low HL in adolescence increases the chance of improper health status that reduces health-promoting behaviour, especially concerning nutrition^(5)^.

As an important aspect of HL, nutrition literacy (NL) is defined as an individual’s ability to access, understand, interpret and apply basic information and services related to nutrition for health promotion^(6,7)^. Developed based on HL theories, NL consists of three sub-sections: functional nutrition literacy, interactive nutrition literacy and critical nutrition literacy^(8)^. Functional nutrition literacy refers to the basic reading and writing skills necessary to understand and follow simple nutrition messages, while interactive nutrition literacy refers to the cognitive and interpersonal communication skills required to receive nutrition information and communicate appropriately with nutrition consultants. Critical nutrition literacy is defined as the ability to critically analyse nutritional information, raise awareness about nutrition and participate in studies aimed at solving nutritional barriers^(8,9)^.

Promoting healthy eating behaviours in adolescents is important for correct growth and development, prevention of disease, prevention of overweight and obesity, and creation of healthy eating patterns that can be maintained in adulthood^(1)^. Healthy eating behaviours are multifactorial; an important overlooked contributor may be NL. While the research literature in NL is growing, it is nonetheless small, requiring the inclusion of general HL literature within discussions of NL^(6)^.

In previous researches, fat intake and unhealthy food consumption were negatively correlated with nutrition-based HL^(5)^. Individuals with low HL levels have difficulty in applying the dietitian’s recommendations correctly^(10)^. In a study, it was determined that individuals with low HL levels were fed unhealthy and those with sufficient HL level preferred fresh, shelled fruits^(11)^. Von Wagner *et al.* reported that individuals with high HL levels perceived themselves as healthy and consumed enough fruits and vegetables^(12)^. Also, according to the Healthy Eating Index, a tool to show the degree of healthy nutrition and diet quality, most individuals were not eating healthy^(10)^. In a study conducted with university students, it was determined that the nutritional literacy score was moderate and the score of women was higher^(13)^. Investigating the relationship between NL and the quantity and quality of dietary intake among youths can aid in the development of effective strategies for promoting nutritional health in this critical age group^(5)^.

In this study, it was aimed to determine the status of nutritional literacy and its affecting factors among the adolescents in Çivril, in Denizli province, in Turkey.

## Methods

Çivril is the largest semi-rural area of Denizli province, in Turkey. During the 2017–2018 academic year, students from ten public high schools in this area formed the universe (*n* 570) of this cross-sectional study. The number of people to be reached was estimated to be 230 using a sample estimation formula for a known universe. It was planned to reach the whole universe without selecting any sample.

The researchers used a face-to-face interview technique to answer the questionnaire form. The questionnaire consists of two parts; Personal Information Form and Adolescent Nutrition Literacy Scale were used as the data collection tool. In the Personal Information Form prepared by the researchers under the literature^(8,9,14)^, there were twenty-four questions about the sociodemographic characteristics, nutritional information, attitudes and behaviours of the participants. Besides sociodemographic characteristics such as age, gender, body weight, height, parent profession and parent education status, there were questions about nutritional information, attitudes and behaviours such as consumption of main meals and snacks, food consumed in school, fast-food consumption frequency, level of confidence in nutrition information sources and nutritional barriers.

In the survey form, the ‘foods consumed during school time’ were grouped as healthy food and unhealthy food. The study of Ng *et al.* ^(15)^ was taken as a guide for making this classification. Healthy foods (core foods), where nutrient content is intense, energy content is low and daily consumption can be recommended; unhealthy foods (non-core foods) contain high amounts of unwanted nutrients such as high fat, salt and refined sugar. Healthy foods were classified as milk, yogurt, buttermilk, water, tea (sugar-free), coffee (sugar-free), fruits, vegetables, salad, nuts, etc. and unhealthy foods, toast, burgers, sandwiches, sugary foods and drinks (chocolate, biscuits, ice cream, candy bars, bar, etc.), chips, crackers, etc^(15)^. Those who consume any of the healthy food groups are evaluated as ‘consuming healthy food’, while those consuming any of the unhealthy foods are ‘consuming unhealthy food’^(15)^.

The Adolescent Nutrition Literacy Scale was created by Bari (2012)^(8)^. Sonay Turkmen *et al.* (2017)^(16)^ adapted it to Turkish. The Turkish adaptation of the Adolescent Nutrition Literacy Scale consists of twenty-two items in the five-point Likert type. The lowest score that can be scored from the scale is 22, the highest score is 110. Increased level of NL provides the higher scores of adolescents^(16)^. Sub-dimensions are:

Functional nutrition literacy: There are seven questions in this section, and the questions in this section are reverse-coded. The minimum score that can be obtained from this sub-dimension is 7 and the maximum score is 35.

Interactive nutrition literacy: This sub-dimension consists of six questions. In this sub-dimension, there is no reverse-coded substance. The minimum score is 6, and the maximum score is 30.

Critical nutrition literacy: There are nine questions in this sub-dimension, three of them (items 18, 19 and 21) are reverse-coded. The minimum score that can be obtained from the sub-dimension is 9, and the maximum score is 45.

The reliability and validity of the ANLS scale in Turkish were performed in adolescents aged 13–16 years^(16)^. Ninth-grade students for high school whose starting age is 15–16 years in Turkey were included in the study.

In our research, age and gender-specific BMI percentile curves were used in the evaluation of BMI. BMI percentile curves were described as obese (percentile ≥ 95), overweight (between 85 and 95 percentile), normal weight (between 5 and 85 percentile) and underweight (≤5 percentile)^(17)^. In this study, body weight and height information were not measured by the researchers, but data were obtained according to the statement of the individuals who participated in the study. Since the distance between the study centre and high schools is about 100 km and the ten different high schools where the study is conducted are also far from each other, weights and stadiometers could not be carried for anthropometric measurements.

The data obtained were evaluated with SPSS 17.0 programme. Where appropriate, *χ* ^2^ test, *t* test in independent groups, one-way ANOVA, Tukey test and linear regression analysis were used in the evaluations.

## Results

A total of 523 (91·7 %) adolescents participated in the study and 49·7 % were female, 47 % in normal BMI and 68·1 % non-smokers. The mean (sd) NL score was 67·6 (sd 7·9). Body perception 58·7 % of participants are ‘frequent/always’ positive and 30·4 % are ‘sometimes’ positive. As the positive body perception increased, NL scores increased and this difference was statistically significant (*P* = 0·03). The difference in positive body perception is between the ‘never/very rarely’ and ‘often/always’ groups (*P* = 0·025). Most (71·9 %) of the participants indicated their health perception status as ‘good/very good’ and 21·8 % as ‘moderate’ (*P* = 0·02). The statistical difference in NL scores resulted from the difference between the health perception ‘very bad/bad’ (63·93 (sd 10·03)) and ‘good/very good’ (67·62 (sd 7·98)) answers (*P* = 0·015). Half (46·1 %) of the participants’ mothers were primary school graduates, and 30·4 % of the participants’ fathers were secondary school graduates. The maternal education and NL levels were positively correlated (*P* = 0·028). The difference in maternal education was between ‘illiterate’ and ‘middle school graduate’ (*P* = 0·03) (Table 1).

**Table 1:** Evaluation of the status of nutritional literacy according to sociodemographic characteristics of students

Variables	*n*	%	NL Score		*P* values
			Mean	sd	
NL Score	523	100	67·62	7·98	–
Gender					0·24
Female	260	49·7	68·03	8·47	
Male	263	50·3	67·22	9·00	
BMI					0·93
Underweight (<5 percentile)	13	2·5	66·46	6·00	
Healthy weight (≥5–<85 percentile)	351	67·1	67·63	8·36	
Overweight (≥85–<95 percentile)	108	20·7	67·87	6·78	
Obese (≥95 percentile)	51	9·8	67·62	7·98	
Smoking status					0·072
Yes	78	14·9	65·71	8·45	
MQuit	89	17	67·83	7·26	
No	356	68·1	67·98	8·01	
Positive body perception					0·03*
Never/Rarely	57	10·9	65·14	9·7·81	
Sometimes	159	30·4	67·52	7·80	
Frequently/Always	307	58·7	68·13	7·98	
Health perception					0·02*
Very Bad/Bad	33	6·3	63·93	10·03	
Middle	114	21·8	67·52	7·19	
Good/Very Good	376	71·9	67·62	7·98	
Maternal educational status					0·028*
Not literate	11	2·1	61·18	9·56	
Literate	5	1	66·40	9·01	
Primary school graduate	241	46·1	67·05	8·32	
Secondary school graduate	169	32·3	68·66	7·43	
High school graduate	77	14·7	67·62	7·69	
College-Faculty Graduation	20	3·8	69·50	6·66	
Father’s educational status					0·69
Not literate	3	0·6	62·67	9·45	
Literate	4	0·8	66·25	6·23	
Primary school graduate	169	32·3	67·07	8·47	
Secondary school graduate	159	30·4	67·66	7·82	
High school graduate	155	29·6	68·12	7·92	
College-Faculty Graduation	33	6·3	68·45	6·55	

* The data are shown as mean values and standard deviations. The one-way ANOVA result was *post hoc* Tukey test which found that the difference in positive body perception was between ‘never/ very rarely’ and ‘often/ always’ (*P* = 0·025), the difference in health perception was between ‘very bad/bad’ and ‘very good/good’ (*P* = 0·015) and the difference in maternal education was between ‘illiterate’ and ‘middle school graduate’ (*P* = 0·03).

When the food consumed in school is evaluated; 51·2 % of the students consume healthy food and 83·7 % consume unhealthy food. The NL scores of those who consume unhealthy foods (67·15 (sd 7·80)) are lower than those who do not consume unhealthy foods (70·04 (sd 8·50)) (*P* = 0·002). While 47 % of the participants visited fast-food restaurants 2–3 times/week, there was no relationship between the frequency of visits to fast-food restaurants and NL scores (*P* > 0·05). 76·5 % of the students consumed three or more main meals a day, 35·9 % of them consumed one snack a day and no correlation was observed between the number of main meals and/or snacks and NL score (*P* > 0·05). The daily water consumption of 60·2 % of the participants was insufficient. The NL scores of adolescents whose water consumption is adequate and inadequate are similar (*P* > 0·05) (Table 2).

**Table 2:** Evaluation of the status of nutritional literacy according to nutritional habits

Variables	*n*	%	NL score		*P* values
			Mean	sd	
Healthy food consumption status					0·24
Yes	268	51·2	67·21	7·09	
No	255	48·8	68·01	8·74	
Unhealthy food consumption status					0·002*
Yes	438	83·7	67·15	7·80	
No	85	16·3	70·04	8·50	
Visiting fast food restaurants					0·5
Everyday	63	12	67·92	7·89	
2–3 times a week	248	47·4	67·36	7·85	
1 time per week	47	9	68·31	7·26	
Once a month	28	5·4	65·46	6·53	
2–3 times a month	15	2·9	70·06	12·01	
Rarely	50	9·6	68·78	11·9·07	
Never	72	13·8	67·33	7·71	
Number of main meals					0·32
1–2 meals	123	23·5	67·00	9·45	
3 meals and over	400	76·5	67·81	7·47	
Number of snacks					0·7
0 meals	131	25	67·75	7·86	
1 meal	188	35·9	67·09	7·66	
2 meals	130	24·9	68·01	7·65	
3 meals and over	74	14·2	68·05	9·50	
Water consumption					0·52
<8 Water glasses†	315	60·2	67·44	7·89	
≥8 Water glass	208	39·8	67·89	8·13	

* *P* < 0·05.

† 1 water glasses = 200 ml.

When the confidence level of the participants was questioned about the accuracy of the nutrition, diet and nutrient knowledge they received, it was determined that 49·5 % were ‘slightly trusted’ and 20·1 % were ‘very sure’. When the barriers to the searching for information about nutrition, diet or nutrients were evaluated, 41·1 % indicated ‘never disagree/disagree’ about the ‘It’s a lot of effort to get the information’ option; 40·1 % specified ‘never disagree/disagree’ about the ‘It is difficult to verify the credibility of the information’ option; 57·6 % indicated ‘agree/completely agree’ on the ‘it is hard to understand the information’ option and 49·5 % said ‘agree/completely agree’ on the ‘it takes a lot of time to search for information’ option. When the level of trust of the participants in the sources of information about nutrition, diet or food was questioned, participants were ‘totally trusted’ to doctors, nurses, and other health professionals (54·5 %), nutritionist, and/or dietitian (53·8 %), textbooks (35·0 %), Internet (34·2 %) and television (36·1 %) (Table 3).

**Table 3:** Evaluation of the status of nutritional literacy according to nutritional information and nutrition information sources

Variables	*n*	%	NL Score		*P* values
			Mean	sd	
Nutritional information accuracy					0·019*
Totally sure	61	11·7	68·26	10·79	
Very sure	105	20·1	69·01	7·18	
Slightly trusted	259	49·5	67·51	7·38	
Very low trust	66	12·6	67·15	7·59	
Never trusted	32	6·1	63·65	7·98	
Internet searching engines					
‘It’s a lot of effort to get the information’					0·019*
Completely disagree/disagree	215	41·1	68·76	8·35	
Undecided	165	31·5	65·99	6·80	
Agree/completely agree	143	27·4	67·72	8·17	
‘It is difficult to verify the credibility of the information’					0·89
Completely disagree/disagree	210	40·1	67·94	9·37	
Undecided	162	31	67·57	7·23	
Agree/completely agree	151	28·9	67·51	7·84	
‘The information is difficult to understand’					0·000*
Completely disagree/disagree	107	20·5	69·27	8·37	
Undecided	167	31·9	66·77	7·39	
Agree/completely agree	249	57·6	66·23	7·68	
‘It takes a lot of time to search for information’					0·026*
Completely disagree/disagree	146	28	68·73	8·58	
Undecided	118	22·6	67·02	7·92	
Agree/completely agree	259	49·5	66·64	6·89	
Information sources					
Doctor, Nurse, Other Healthcare professionals					0·067
Very Low/Low Trust	141	27	66·29	8·80	
Undecided	97	18·5	67·92	6·70	
Strongly Trusted/Totally Trusted	285	54·5	68·17	7·91	
Nutritionist, Dietitian					0·001*
Very Low/Low Trust	121	23·1	65·86	8·17	
Undecided	121	23·1	66·59	7·32	
Strongly Trusted/Totally Trusted	281	53·8	68·82	7·98	
Textbooks					0·015*
Very Low/Low Trust	199	38	66·55	7·92	
Undecided	183	35	67·65	7·85	
Strongly Trusted/Totally Trusted	141	27	69·09	8·05	
Television					0·09
Very Low/Low Trust	252	48·2	67·72	8·49	
Undecided	204	36·1	66·84	7·48	
Strongly Trusted/Totally Trusted	51	15·7	69·12	7·29	
Internet					0·35
Very Low/Low Trust	244	46·7	67·08	8·11	
Undecided	179	34·2	68·09	7·22	
Strongly Trusted/Totally Trusted	100	19·1	68·10	8·90	

* The data are shown as mean ± SD. The one-way ANOVA result was *post hoc* Tukey testing and found that the difference in article ‘nutritional information accuracy’ was between ‘very sure’ and ‘never trusted’ (*P* = 0·014). The difference in the clause ‘It’s a lot of effort to get the information’ was found to be between ‘never disagree/disagree’ and ‘undecided’ (*P* = 0·014). The difference in answers to the sentence ‘The information is difficult to understand’ was found to be between ‘never disagree/disagree’ and ‘undecided’ (*P* = 0·007) and between ‘never disagree/disagree’ and ‘agree/ completely agree’ (*P* = 0·001). The difference in the answer ‘it takes a lot of time to search for information’ was found to be between ‘never disagree/disagree’ and ‘agree/ completely agree’ (*P* = 0·040). The difference in the answer ‘nutritionist. dietician’ was found to be between ‘very low/low trust’ and ‘strongly trusted/totally trusted’ (*P* = 0·002) and between ‘undecided’ and ‘strongly trusted/totally trusted’ (*P* = 0·026). The difference in the answer ‘textbooks’ was found to be between ‘very low/low trust’ and ‘strongly trusted/totally trusted’ (*P* = 0·001).

When the relationship between the confidence level and accuracy of the information obtained from the participants about nutrition, diet and food, NL scores were examined; the scores of those who were very sure (69·01 (sd 7·18)) were higher than those of those who never trusted (63·65 (sd 7·98)) (*P* = 0·014). When the relationship between information barriers and NL score of adolescents about nutrition, diet and food was questioned, the difference in ‘It’s a lot of effort to get the information’ was found to be between ‘never disagree/disagree (68·76 (sd 8·35)’) and ‘undecided (65·99 (sd 6·80))’ (*P* = 0·014). The difference in the clause ‘it takes a lot of time to search for information’ was found to be between the clause ‘never disagree/disagree (68·73 (sd 8·58))’ and the clause ‘agree/completely agree (66·64 (sd 6·89))’ (*P* = 0·040) (Table 3).

When the relationship between the level of trust in nutrition information sources and the NL score of the participants was examined, the difference in ‘nutritionist, dietician’ was found to be between ‘very low/low trust (65·86 (sd 8·17))’-‘strongly trusted/totally trust (68·82 (sd 7·98))’ (*P* = 0·002) and ‘undecided (66·59 (sd 7·32))’-‘strongly trusted/totally trust (68·82 (sd 7·98))’ (*P* = 0·026). The difference in the‘ textbooks ’category was determined to be between ‘very low/low trust (66·55 (sd 7·92))’ and ‘strongly trusted/totally trust (69·09 (sd 8·05))’ (*P* = 0·001). Other data on distribution are presented in Table 3.

The multivariable analysis showed that the NL score of adolescents and their age, gender, smoking status and level of certainty from the accuracy of nutrition-related information were determined to be influenced by variables other than the clause ‘it takes a lot of time to search for information’. Maternal educational status, health perception, positive body perception, unhealthy food consumption, efforts required to learning information, the difficulty level of understanding information, trust levels to the nutritionist/dietitian and trust levels to textbooks were related variables to the NL scale score (*P* < 0·05) (Table 4).

**Table 4:** Factors affecting nutrition literacy

Variables	B	SE	CI	*P*
Constant	41·4	5·3		
Maternal Educational Status (from no literate to faculty graduation)	1·5	0·6	0·2, 2·8	0·021*
Health Perception (from bad to good)	3·7	1·4	0·9, 6·4	0·008*
Positive Body Perception (from never to always)	2·3	1·0	0·2, 4·4	0·032*
Unhealthy Food Consumption (from yes to no)	−2·2	0·9	–4·0, –0·3	0·017*
Information engines				
Undecided about ‘It’s a lot of effort to get the information’ (among others)	1·8	0·8	0·2, 3·3	0·026*
Undecided about ‘The information is difficult to understand’ (according to others)	1·6	0·8	0·06, 3·2	0·042*
Thinking about ‘It takes a lot of time to search for information’ (according to others)	2·4	0·8	0·8, 4·05	0·003*
Trust in nutrition, diet information sources				
‘Strongly/Totally’ to trust nutrition and diet expert, dietitian (according to others)	2·7	0·8	1·09, 4·35	0·001*
‘Undecided’ to trust nutrition and diet expert, dietitian (compared with others)	2·13	0·82	0·4, 3·7	0·011*
‘Strongly/Totally’ to trust the textbooks (according to others)	1·7	0·7	3·2, 0·2	0·023*

* Age, gender, smoking status, level of certainty from the nutritional information accuracy. ‘it takes a lot of time to search for information’ was also added to the model. Linear regression (enter) analysis was done and was not statistically significant (*B* = Regression Coefficient) (*R* ^2^ = Correlation Coefficient).

## Discussion

In this study, adolescent NL was found to be moderate. In similar research with adolescents and university students, the level of nutritional literacy is also moderate^(8,13,18)^. In another study to determine the NL level of adults in the Mississippi Delta, 48 % of participants were found to have sufficient NL^(19)^. According to another study (*n* 376), only 26 % of respondents had adequate HL levels and the one-point increase in HL score was also associated with a 1·21-point increase in the Healthy Eating Index (HEI) score, which is one of the most basic indicators of healthy eating^(20)^.

In the European HL study (*n* 7795)^(2)^ and the Turkish Health Literacy Scale (TSOY-32)^(21)^, HL was found to be moderate (*n* 400). The Health and Social Workers Union conducted a study to assess the HL standard, and 64·6 % of the participants were found to be ‘inadequate’ or ‘problematic’^(22)^. In another study conducted abroad (*n* 508), 73 % of the participants were found to be ‘inadequate’ HL^(23)^. In a research with medicine and nursing students, one-third of students have a restricted and inadequate level of HL^(24)^. According to the results of the National Assessment of Adult Literacy Study conducted with 19 000 participants, 12 % of US adults have adequate and 53 % have moderate HL^(19)^. According to the research of HL levels, 70·8 % of respondents had a low level of literacy and only 11 % had a high level of literacy^(25)^. The study, which was conducted with the participation of students from Health Sciences and Social Sciences, found that only 6 % of students were at an excellent HL level and that the health student score was higher^(26)^.

Each level of maternal education was determined to have increased to NL by about 1·5 points. As mothers education level increases is more likely her children know nutrients, and therefore to prefer healthy and low-energy-intensive foods, and these results show consistency^(27)^. In contrast to our research, there was no significant relationship between mother education and nutritional literacy^(18)^. One research demonstrates that adolescent fruit and tea intake decreased as maternal education increased and three meals a day nutrition increased^(28)^. A study of forty-eight parents and children showed a poor correlation between parental NL and child dietary quality^(29)^.

In this study, a relationship between the health perception of adolescents and the NL score was determined. Demir Özdenk and Özcebe (2018) found no significant relationship between perception of health and nutritional literacy^(30)^. A bad health perception is a risk factor for negative eating attitudes of high school students^(31)^. A study using three different HL and health perception scales found a positive correlation between HL and health perception^(32)^. According to the results of the European HL study, it was determined that individuals with ‘very bad/ bad’ health perception had limited HL levels^(2)^. There was no relationship between nutritional literacy and general health perceptions among high school students^(18)^.

In our study, a relationship between positive body perception and NL score was determined. A similar study found no relationship between physical appearance satisfaction and nutritional literacy among high school students^(18)^. In a study conducted with the participation of adolescents (*n* 2146), unhealthy eating behaviours were found to be strongly associated with higher BMI and poor body perception in both sexes^(33)^. A positive correlation was found between high school students’ body perception and eating attitudes^(34,35)^.

According to this study, the NL score of adolescents consuming unhealthy foods at school was found to be 2207 points lower than those who did not consume it. A similarly conducted study found that individuals who consumed sugary food had lower HL and HEI scores^(19)^. In another study, it was determined that every one-point increase in HL decreased the consumption of sugary foods and drinks by 34 kcal/d^(20)^. In a study of 360 adolescents conducted by Williams and Mummery in Australia in 2012, it was found that while adolescents are aware of the rules of healthy eating, many of them consume unhealthy foods such as high-fat fast food and participate in unhealthy habits such as skipping meals to lose weight^(36)^.

In our study, there was a significant relationship between some information barriers (effort required to learn information, difficulty level of understanding information) and NL. In contrast to our study, a study in which similar questions were questioned found no statistically significant relationship between information barriers and the NL^(8)^. Zoellner *et al.* observed that adults with lower nutritional literacy rated nutritional knowledge barriers higher than those with sufficient literacy, but the pattern was not important^(19)^. In another study, the relations between functional and critical NL and barriers to finding information on food and nutrition were considerably negative^(5)^.

In this study, it was found that the NL score increased and was statistically significant as adolescents increased confidence in nutrition information from nutritionist/dietitian and textbooks. In a similarly conducted study, half of the adolescent students stated that they relied on nutritional information obtained from nutritionists or dietitians. This suggests that nutritionists and dietitians may play an important role in influencing how adolescents receive their nutritional information and thus affect their eating habits^(8)^.

According to data from the 2018 Food and Health Study (*n* 1009), when the sources that participants rely on about which food to eat or avoid are questioned, similar to the 2017 results of the same study, the expert dietitian and/or nutritionist and dietitian are ranked first^(37)^. In Cash *et al.*’s study, dietitians, nutritionists and general physicians were three most preferred sources and were perceived to be the most trustworthy, credible and effective^(5,38)^.

The study examined the level of trust in nutrition information sources; the family (57·2 %) was the most reliable source followed by doctors and other health workers (54·5 %) and nutritionist/dietitian (53·8 %), while the least reliable source was radio (10·1 %)^(39)^. In the Nutrition and You: Trends 2011 study conducted by the American Dietetic Association, it was found that television (62 %) was the most popular source of nutrition information in individuals aged 18–24 years, followed by the Internet (46 %) and magazines (29 %)^(40)^.

Zoellner *et al.* according to their study, participants relied most on the information they gained from doctors or health care providers and television, while they relied least on information they gained from the Internet^(19)^. According to a study, the majority of adolescents said they relied heavily on nutrition information from international organisations such as the WHO and strongly relied on resources such as a nutritionist or dietitian (55 %), a doctor or nurse (45 %), while the least trusted on nutrition information received from friends^(8)^.

A study found that 77·0 % of 14–21 age group teens use the Internet to learn about their health and that Internet use is greater in adolescents, and this use is likely to increase in health. According to another study, among the participants, the most common source of nutrition and dietary information was television (79·8 %), newspapers (57·8 %) and books/magazines (41·4 %). Nutrition and dietary information sources are associated with adequate NL^(41)^.

In the study in Uganda, about a third of adolescent students stated that the Internet is a source of nutrition information. From an adolescent perspective, the Internet is seen as a powerful resource in health information and usage. The use of the Internet as a source of health information has also led some adolescents to change their behaviour and seek health care^(8)^.

Another study on adolescents found that 25·0 % of the students received partial nutrition education, 49·2 % did not receive any nutrition education at all and 4·2 % of the nutrition information were from books, and 20·8 % from school^(42)^. In another study, 66·8 % of students reported receiving information about adequate and balanced nutrition, while teachers (29·4 %) and radio-television (26·7 %) were the first two sources of information^(14)^.

In a study with university students, they noted that students listened to programmes about nutrition on radio and television (26·0 %, 54·0 %) and use this information. The proportion of those who read articles about nutrition in the newspapers was 40·5 % and 31·7 % said they used this information^(43)^.

It states that adolescents have greater access to health and nutrition information because of their greater exposure to outside resources, such as the Internet, awareness programmes in schools and social media advertisings^(14)^. In the study, however, more than 70 % of students reported needing nutrition-related information.

### Implications for research and practice

NL was found to be moderate in the study. It has been found that adolescent NL affects maternal education level, health perception, positive body perception, unhealthy food consumption status, some information barriers (effort required to learn information, difficulty level of understanding information) and some level of confidence in information sources (nutritionist and/or dietitian, textbooks).

The NL levels of individuals and communities need to be increased to ensure healthy eating. Health policies should be developed to improve NL. When the projects and programmes related to nutrition are examined in our country, there are no programmes that involve multiple behaviour development initiatives, which span a long time and which have sustainability. In addition to increasing women’s education, it is appropriate to make initiatives in adolescents to increase the perception of body and general health and to move away from the barriers of nutritional information.

Adolescents have insufficient knowledge of NL level and healthy eating. Unhealthy eating habits are also observed. As a result of the study, based on the knowledge that adolescents have a high level of confidence in dietitians, nutrition education should be provided at frequent intervals through community health dietitians to ensure healthy eating. The Ministries of Health and Education must cooperate in this regard. Appropriate technology applications to healthy eating programmes should also be added to the curriculum as a course.

Studies on the NL are inadequate. Addressing this issue by experts and improving nutritional literacy is important for community health.

This study was conducted to determine the status of adolescent nutritional literacy and to determine the factors affecting nutritional literacy. Reaching 91·7 % of the universe is one of the strengths of this research. During the study, maximum attention was paid and ethical rules were adhered to at every stage. A significant contribution was made to the literature with the statistical data obtained. For the data obtained within the scope of our research to be evaluated more broadly, new studies are needed.

In this study, the lack of food consumption records and insufficient anthropometric measurements are among the important limitations. Transportation problems between the study centre and high schools made it difficult to transport the devices to be used for anthropometric measurements. The lack of a sufficient number of trained researchers to obtain food consumption records made it difficult to obtain these records. Similarly, issues such as school and environmental health, advertisements, were not addressed. It is useful to look at subsequent studies.

## Author Note

This study is derived from a master’s thesis entitled Çağla AYER ‘Çivril Yöresindeki Adolesanlarda Beslenme Okuryazarlığının Mevcut Durumu ve Etkileyen Faktörler’ subject which was conducted in the Department of Public Health of Pamukkale University Institute of Health Sciences which was consulted by Prof. Dr. Ahmet ERGİN.

This work was presented as a poster paper at the ‘1st International Health Science and Life Congress’ from May 2–5, 2018, and printed in the abstract booklet.

This work was presented as an oral statement at the ‘1st International Food & Medicine Congress’ from May 24–27, 2018, and printed in the abstract booklet.
